# Supplementary material for: Time‑resolved multi-omic analysis of paclitaxel exposure in human iPSC‑derived sensory neurons unveils mechanisms of chemotherapy‑induced peripheral neuropathy
Source: Cell Death Dis. 2026 Feb 10;17(1):211. doi: 10.1038/s41419-026-08445-2 (PMC12921266; doi:10.1038/s41419-026-08445-2)
Supplement: Supplementary file 15 — Dataset 1. [file 41419_2026_8445_MOESM15_ESM.zip › CDD_Open_Data/04_RNA_Seq/Timeline_panel_plots_etc_2023-01-23_1609.zip!/Panel_Plots_etc/Evidence_Plots/post5d/evidence_plot_standard contrasts_msigdb_hallmark_5d_PTX_vs_DMSO_ID6_M5902_HALLMARK_APOPTOSIS.pdf]

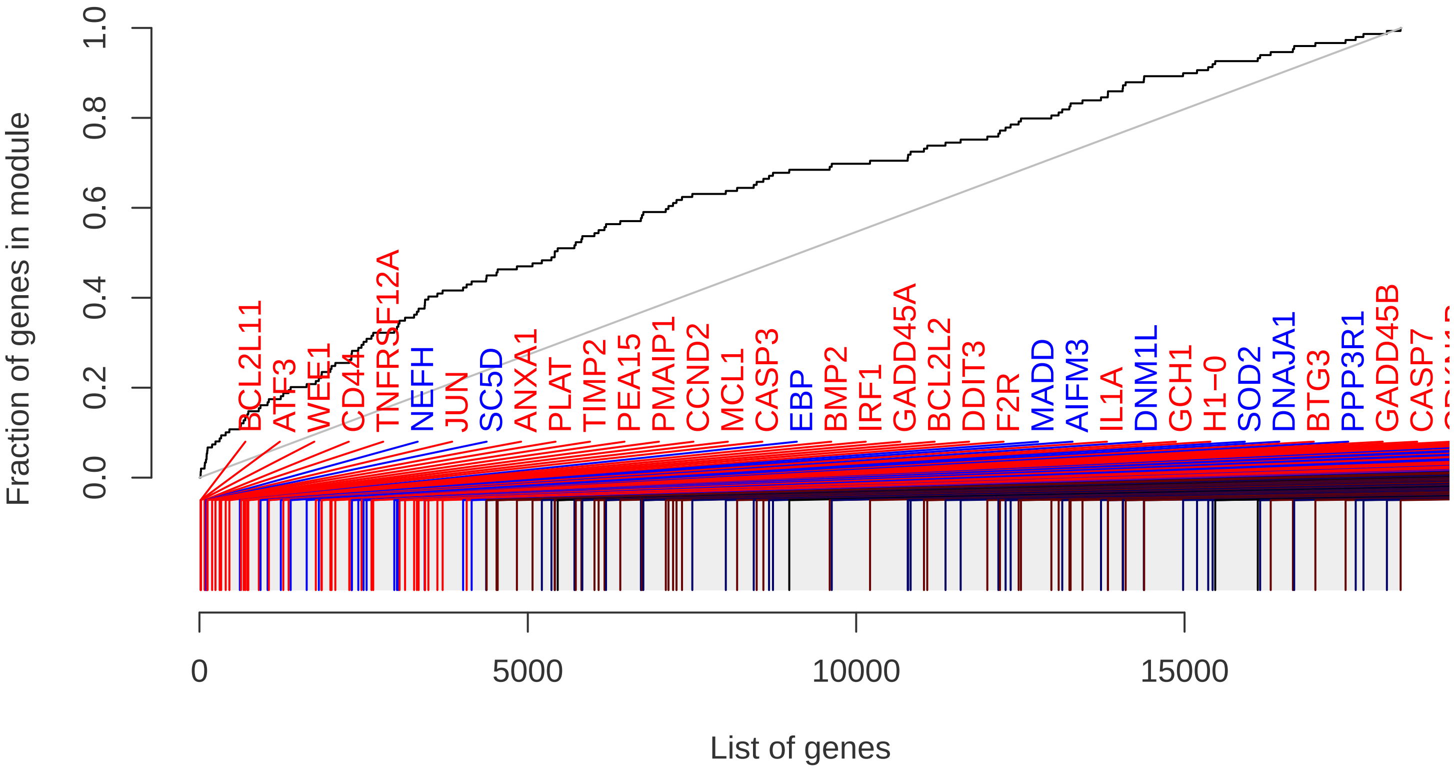

Fraction of genes in module
0.0
0.2
0.4
0.6
0.8
1.0
BCL2L11
ATF3
WEE1
CD44
TNFRSF12A
NEFH
JUN
SC5D
ANXA1
PLAT
TIMP2
PEA15
PMAIP1
CCND2
MCL1
CASP3
EBP
BMP2
IRF1
GADD45A
BCL2L2
DDIT3
F2R
MADD
AIFM3
IL1A
DNM1L
GCH1
H1−0
SOD2
DNAJA1
BTG3
PPP3R1
GADD45B
CASP7
0
5000
10000
15000
List of genes
